# Chemical probe mediated visualization of protein S-palmitoylation in patient tissue samples

**DOI:** 10.3389/fphys.2023.1063247

**Published:** 2023-02-21

**Authors:** Nancy Schek, Jia-Ying Lee, George M. Burslem, Eric Witze

**Affiliations:** ^1^ Department of Cancer Biology, Philadelphia, PA, United States; ^2^ Perelman School of Medicine, University of Pennsylvania, Philadelphia, PA, United States; ^3^ Department of Biochemistry and Biophysics, Philadelphia, PA, United States; ^4^ Abramson Family Cancer Research Institute, Philadelphia, PA, United States

**Keywords:** palmitoylation, cancer, signaling, chemical probe, EGFR

## Abstract

While protein palmitoylation has been studied for decades, our understanding of its clinical importance is minimal compared to other post translational modifications. As a result of the inherent challenges preventing the production of antibodies to palmitoylated epitopes we are unable to correlate levels of protein palmitoylation in biopsied tissues at a meaningful resolution. The most common method for detecting palmitoylated proteins without metabolic labelling is through chemical labeling of palmitoylated cysteines with the acyl-biotinyl exchange (ABE) assay. We have adapted the ABE assay to detect protein palmitoylation in formalin fixed paraffin embedded (FFPE) tissue sections. The assay is sufficient to detect subcellular regions of cells with increased labeling which indicates areas enriched in palmitoylated proteins. To visualize specific palmitoylated proteins in both cultured cells and in FFPE preserved tissue arrays we have integrated the ABE assay with a proximity ligation assay (ABE-PLA). Our findings demonstrate for the first time that FFPE preserved tissues can be labelled with unique chemical probes to detect either areas enriched in palmitoylated proteins or the localization of specific palmitoylated proteins using our ABE-PLA methodology.

## Introduction

Chemical probes enable detection of the chemical reactivity of proteins and enable activity-based proteomics screens to identify reactive proteins in cell lysates and potential drug binding sites on proteins (Bak et al., 2019), (Adam et al., 2002). However, methods to determine the spatial localization of the reactive form of the protein in intact cells under specific environmental conditions and more importantly in patient tissue samples are still lacking (Lu and Fang, 2020). An essential application for chemical probe labeling is in detecting protein modifications that are not recognized by conventional antibody-based methods and require chemical labeling of the modified residue. For example, S-palmitoylation is a common protein modification, but its physiological and disease importance is still poorly understood since it is difficult to directly measure protein palmitoylation in disease tissues. While an antibody to palmitoylated postsynaptic scaffold protein PSD-95 has been reported, the hydrophobic properties of palmitate have hindered production of antibodies which are typically used to correlate PTMs like phosphorylation and acetylation with disease state in patient samples thus new chemical biology approaches are required (Fukata et al., 2013; Main and Fuller, 2022).

**FIGURE 1:**
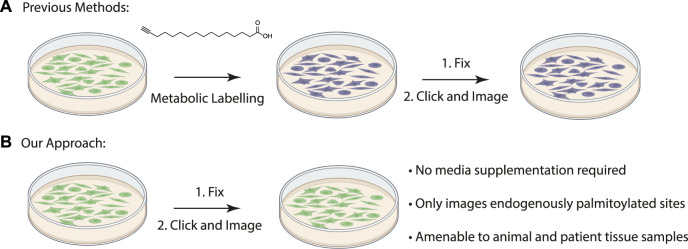
**(A)** Schematic of previously described procedure for visualizing palmitoylated proteins by metabolic labeling of cultured cells with palmitic acid alkyne. **(B)** New method to visualize palmitoylated proteins without metabolic labeling.

Duolink proximity ligation assay generates a fluorescent signal when the two antibodies are within 40 nM from each other (Fredriksson et al., 2002). Previously proximity ligation assays have been used to visualize specific palmitoylated proteins in cells by metabolically labeling cultured cells with palmitic acid alkyne followed by Cu(II) mediated azide alkyne cycloaddition (CuAAC) Oregon green-azide (Figure 1A) (Rostovtsev et al., 2002; Tornøe et al., 2002; Gao and Hannoush, 2016). Protein specific antibodies are used to label the target protein and Oregon Green is detected with anti-Oregon Green antibodies. This method allowed for the first time the visualization of subcellular localization of the O-palmitoylated proteins hedgehog and Wnt as well as S-palmitoylated Ras, but its use is limited to cultured cells that can be metabolically labelled (Gao and Hannoush, 2016). Visualizing palmitoylated proteins in patient samples requires methods free of metabolic label detection that have yet to be described.

The acyl-biotinyl exchange (ABE) assay is a method for detecting palmitoylated proteins in cell lysates which uses cysteine reactive probes to label palmitoylated cysteine residues (Drisdel and Green, 2004). The free cysteine thiols are blocked with n-ethylmaleimide (NEM) after which palmitate is removed with hydroxylamine and the revealed free cysteine residues are labeled with a thiol reactive agent such as iodoacetamide-biotin or biotin-HPDP (N-[6-(biotinamido) hexyl]-3’-(2′-pyridyldithio) propionamide) (Figure 1B). The advantage to this method is that unlike metabolic labeling with click-based probes the ABE is amenable to detecting proteins in formalin-fixed, paraffin-embedded (FFPE) patient samples. An assay for measuring palmitoylation of specific proteins in patient samples will allow direct correlations between palmitoylation levels of specific proteins and disease state. As a first step we asked if chemical probes react with target proteins in FFPE prepared tissue samples allowing us to detect S-palmitoylated proteins. We sought to extend this method to the chemical labeling of specific proteins through the integration of the ABE assay with a proximity ligation assay (PLA) in FFPE tissue samples and thus demonstrate for the first time that chemical imaging of PTMs is possible in fixed tissues.

## Results and discussion

### Fluorescent imaging of ABE labeled palmitoylated proteins in FFPE samples

In the ABE assay free cysteine thiols are blocked with N-ethylmaleimide (NEM) to prevent non-specific labeling of unpalmitoylated cysteines with the cysteine reactive probe. We anticipated that the main challenge in labeling FFPE tissues is saturating the free thiols with NEM in the highly crosslinked fixed tissues to reduce non-specific background labeling and therefore carefully optimized a procedure for efficient ABE in fixed tissues.

**FIGURE 2:**
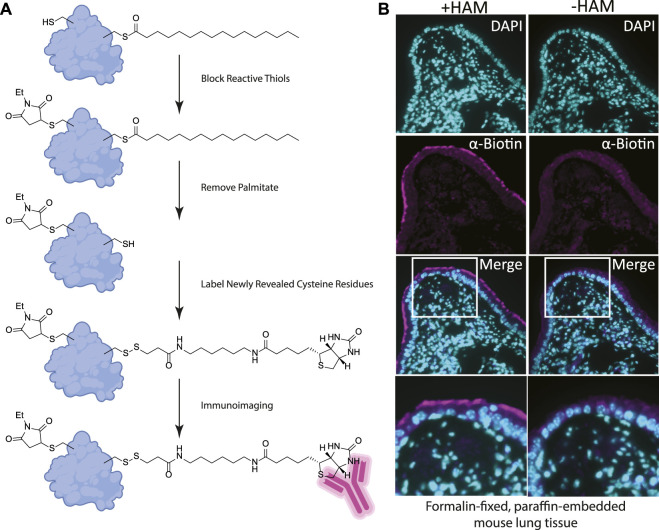
**(A)** Label free method for detecting palmitoylated proteins to be used on FFPE prepared tissues. 1) NEM treatment blocks free thiol groups on all proteins. 2, 3) Hydroxylamine (HAM) treatment removes palmitate and biotin-HPDP reacts with the revealed thiol. As a negative control HAM is omitted, but biotin-HPDP is still added. 4) The biotin is recognized with an anti-biotin antibody and detected with a fluorescently conjugated secondary antibody. **(B)** FFPE tissue sections of mouse lung airway. The hydroxylamine treated (+HAM) samples reveal anti-biotin antibody labelling at the apical surface of the airway cells (magenta). In an adjacent section of the same sample where hydroxylamine (HAM-) has been omitted the labeling is absent. DNA is stained with DAPI (cyan).

We reasoned that for the ABE assay to detect palmitoylation of unidentified proteins in FFPE samples we would need to identify cells enriched in palmitoylated proteins. Using FFPE mouse lung serial sections as a model system, we prepared them as for standard immunohistochemistry. Sections were treated with xylene to solubilize and remove all paraffin, followed by washes in ethanol to remove the xylene, and then in decreasing concentrations of ethanol for rehydration. In order to remove protein crosslinks formed by formaldehyde treatment, which can mask antibody-binding epitopes, sections were heated to 100°C (Kim et al., 2016). Immediately following this antigen retrieval step, slides were blocked with buffer containing NEM in 150 mM NaCl, 50 mM HEPES pH 7.4, 5 mM EDTA (ABE buffer), and 0.2% Triton X-100. After washing with ABE buffer, slides were incubated with 1.5 M solution of hydroxylamine in ABE buffer and as a negative control a slide containing an adjacent serial tissue section was incubated in the same buffer without hydroxylamine. Slides were washed followed by incubation in buffer containing biotin-HPDP (N-[6-(biotinamido) hexyl]-3’-(2′-pyridyldithio) propionamide). The slides were blocked in 5% bovine serum albumin (BSA) dissolved in tris buffered saline with 0.5% Tween 20 (TBST) followed by an overnight incubation with anti-biotin antibody (Figures 1B, 2A). After incubation with fluorescently tagged secondary antibody slides were mounted and imaged by widefield fluorescence microscopy.

Building on our previous studies examining the function of palmitoylated EGFR in lung adenocarcinoma, we chose to use FFPE mouse lung sections to develop the assay (Kharbanda et al., 2020). Gratifyingly, using our optimized procedure, we observed a distinct subcellular labelling pattern in the airway of the lungs with the anti-biotin antibody restricted to the apical surface of the airway epithelial cells (Figure 2B). This labeling was absent in the -hydroxylamine control, validating our method was labeling only palmitoylated (thioester) proteins that are enriched at the apical surface of the airway cells. The results of this *in situ* labeling method are consistent with the important role protein palmitoylation plays in establishing or maintaining polarized structures in cells. Ciliated cells line the airway and a dual-lipidation signal consisting of myristoylation followed by palmitoylation functions as a cilia protein targeting signal (Kumeta et al., 2018). Whilst this demonstrates the chemical probe-based imaging of cysteine palmitoylation in FFPE tissue for the first time, it lacks protein level specificity which is crucial for understanding PTMs. To address this liability, we next asked if this method could be adapted to detect specific proteins that react with the cysteine reactive probe *via* proximity ligation assays.

### ABE-PLA assay in cultured cells

The proximity ligation assay (PLA) generates a signal when the antibody to the labelled cysteine is within 40 nm of the binding site of the protein specific antibody. With the success in detecting protein palmitoylation in FFPE tissues with the ABE assay we sought to integrate the ABE assay with the PLA to detect specific palmitoylated proteins without the need for metabolic labelling, which is impossible in human tissues. Our lab has previously found that the epidermal growth factor receptor (EGFR) is palmitoylated in multiple cancer cell lines and blocking EGFR palmitoylation blocks Kras driven lung tumor growth in animal models (Runkle et al., 2016; Kharbanda et al., 2020). We showed previously that EGF stimulation increases palmitoylation of phosphorylated EGFR (Runkle et al., 2016). We therefore sought to visualize palmitoylated phosphorylated EGFR in H1975 lung cancer cell lines which harbor activating mutations in EGFR.

**FIGURE 3:**
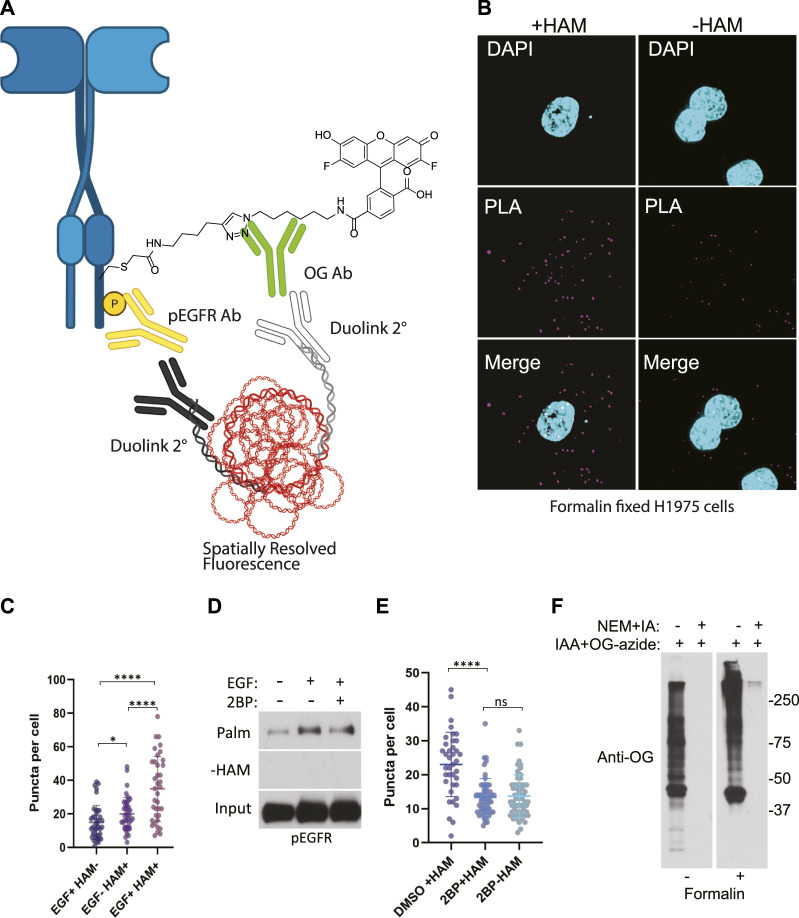
**(A)** Schematic of the ABE-PLA showing method for detecting palmitoylated EGFR in formalin fixed cells processed with the ABE protocol and palmitate was replaced with Oregon green-iodoacetamide. Following the ABE, the proximity ligation assay was performed using primary antibodies to EGFR-phosphoTyrosine1068 and the Oregon Green (OG) label. Samples were incubated with species specific secondary PLA antibodies followed by annealing to single stranded circular DNA which is then amplified with DNA polymerase. The amplified DNA is detected with fluorescently tagged complimentary oligonucleotides and is visualized as fluorescent puncta. **(B)** Formalin fixed H1975 lung cancer cells processed with the ABE-PLA. PLA signal is detected in cells treated with hydroxylamine (+HAM). Omitting hydroxylamine reduces the number of puncta in the negative control (-HAM) DAPI is shown in cyan and PLA signal in magenta. **(C)** Puncta per cell were quantified in all conditions (Unpaired Student’s *T*-test: EGF + HAM-vs. EGF + HAM+ ****p < 0.0001; EGF + HAM + vs. EGF- HAM+ ****p < 0.0001; EGF + HAM-vs EGF-HAM+ *p < 0.05). Total Number of Cells = 119. **(D)** Standard ABE assay of H1975 cells treated with 100 nM 2-bromopalmitate followed by 100 ng/mL EGF stimulation. **(E)** Quantitation of ABE-PLA puncta of cells with or without 2-bromopalmitate with and without EGF stimulation. [Unpaired Student’s *T*-test: DMSO + HAM + vs. 2BP + HAM+ ****p < 0.0001; 2BP + HAM + vs. 2BP + HAM-not significant (ns)]. Total Number of Cells = 153. **(F)** Validation of efficient cysteine blocking and labelling of formalin fixed cells *in vitro*.

We stimulated H1975 cells with EGF for 5 min to increase EGFR auto-phosphorylation compared to non-treated controls. Cells were then fixed in formalin containing NEM and permeabilized with ABE buffer containing 0.2% Triton X-100 and NEM to quench reactive cysteines as before. Coverslips were washed in 0.2% Triton X-100 ABE buffer and incubated in the same buffer containing iodoacetamide which we found during extensive optimization was necessary to block free cysteine residues and reduce non-specific cysteine labeling. Coverslips were washed and incubated with or without HAM followed by incubation with the cysteine reactive label. Initial samples were prepared using biotin-HPDP however, labelling with biotin resulted in high background which could be due to presence of endogenous biotin. Subsequently, we switched to using iodoacetamide-alkyne followed by Cu(II) mediated azide alkyne cycloaddition (CuAAC) with Oregon Green-azide to label the HAM unmasked reactive cysteine residues. Coverslips were then incubated with antibodies to Oregon Green and to phospho-EGFR (Y1068). Finally, the proximity ligation assay (Duolink) was performed by the addition of oligo labelled secondary antibodies followed by a polymerase and fluorescently labelled complementary oligos, and coverslips were mounted and imaged (Figure 3A). The PLA puncta were present in multiple focal planes and therefore a z-series of images were captured allowing quantification of puncta throughout the cell. This results in fluorescent puncta which are significantly more numerous in cells stimulated with EGF and appear in a HAM dependent fashion, thus indicating that they result specifically from EGFR bearing both palmitoylation and phosphorylation PTMs (Figures 3B, C).

While the omission of hydroxylamine significantly decreased the number of puncta compared to the hydroxylamine treated sample, we further confirmed that the puncta are representative of palmitoylated EGFR by treating cells with the palmitoylation inhibitor 2-bromopalmitate (2BP). Using the conventional ABE assay, we detect an increase in palmitoylated EGFR phosphorylated on tyrosine 1068 upon EGF treatment (Figure 3D). Treatment of the cells with 100 nM 2BP decreased palmitoylated EGFR compared to DMSO control but is still higher than -HAM control. Using the ABE-PLA assay, treatment of cells with 2BP reduced the number of puncta compared to cells treated with the vehicle control (Figure 3E). Omitting hydroxylamine did not reduce the number of puncta below cells treated with 2BP (Figure 3E).

Finally, we asked if the puncta present in the -HAM control were the result of incomplete blocking of thiols due to chemical fixation. Cultured cells were fixed with formalin containing NEM, followed by blocking cysteine residues with NEM and IA in ABE buffer while attached to the tissue culture plate. The cells were then washed and treated with iodoacetamide alkyne which was then conjugated to Oregon Green-azide. The cells were then harvested, and lysate was analyzed by SDS-PAGE followed by immunoblotting with anti-Oregon Green antibodies. We found that treating cells with formalin resulted in a very minor increase in background in the NEM/IA treated condition compared to cells that were not formalin fixed (Figure 3F). We concluded that the background PLA puncta were not likely caused by residual free thiols. The background in the fixed cells could be the result of non-specific binding of the labeling agents that may require additional blocking strategies to further reduce the background.

Having developed a novel chemical probe-based imaging approach (ABE-PLA) for palmitoylation, we sought to apply it to the imaging of disease relevant human samples.

### ABE-PLA in FFPE tissue arrays

**FIGURE 4:**
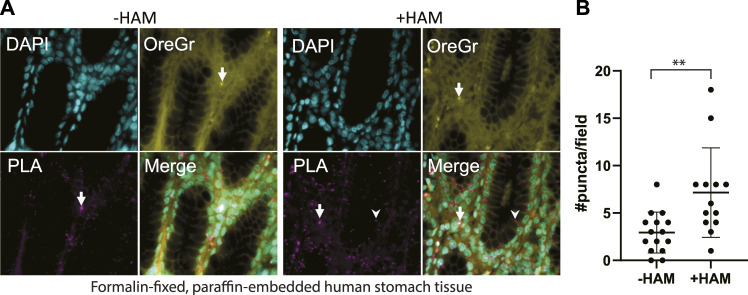
**(A)** Multi-organ tissue arrays were treated with the ABE-PLA. DNA was labelled with DAPI (cyan). Cytosolic staining of cells demarcating the cell boundary is visible in the Oregon Green channel (Yellow). Hydroxylamine treatment increases the number of magenta PLA puncta (arrowhead) compared to the -hydroxylamine control in the epithelia of the stomach tissue samples. **(B)** Quantification of the number of puncta per field in two sample cores. Puncta that are both magenta and yellow (arrow) were not counted. Error bars are standard deviation. (Unpaired Student’s *T*-test **p-value = 0.0045).

We applied our optimized ABE-PLA approach to tissue arrays of multiple human tissue types. In the tissue sections we observed two distinct types of PLA positive puncta. Both types of puncta were present in the HAM treated, as well as the untreated negative control. One type was characterized by magenta PLA probe puncta that appeared in the absence of Oregon Green puncta (Figure 4A, arrowhead). The second type had equally high levels of Oregon Green and magenta PLA probe signal (Figure 4A, arrow). We reasoned that if the signal was the amplified product of the PLA, then the PLA signal should be markedly stronger than any non-specific Oregon Green labeling. We therefore only scored a magenta punctum to be PLA positive if the Oregon Green did not form a punctum at the same site. In stomach tissues we observed clear PLA specific puncta that increase in the HAM treated sample (Figure 4B).

**FIGURE 5:**
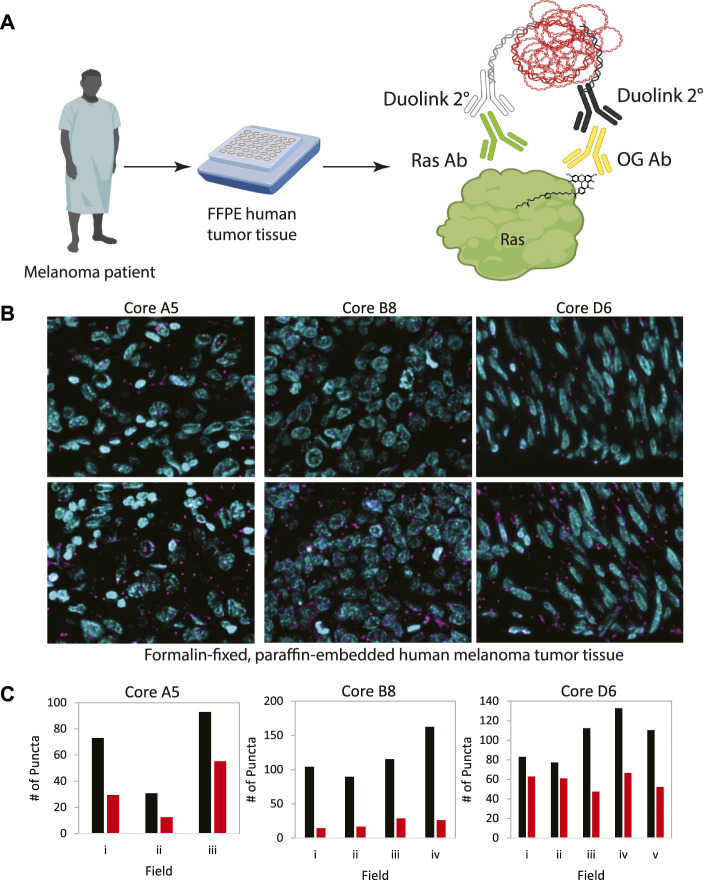
**(A)** Workflow for analysis of patient tumor samples on a tissue array. **(B)** Melanoma tissue arrays were treated with the ABE-PLA. Three different samples are shown. **(C)** The number of PLA puncta (magenta) per field were counted in the -HAM control slide and the +HAM treated slide. Variation in background signal (-HAM, Red bars) between samples and between fields within a sample are shown compared to the ABE generated signal (+HAM, Black bars). Statistical significance of the difference between–HAM and + HAM samples: A5 *p*-value = 0.049, B8 *p*-value = 0.006, D6 *p*-value = 0.015; Paired Student’s *T*-test.

In order to demonstrate the generalizability of this approach to visualize other palmitoylated proteins using the ABE-PLA, we next examined the localization of palmitoylated Ras in human patient melanoma arrays (Figure 5A). While only a small fraction of EGFR is palmitoylated, HRas and Nras rapidly cycle between palmitoylated and unpalmitoylated states which shuttles Ras between the plasma membrane and Golgi apparatus (Dudler and Gelb, 1996; Rocks et al., 2005). Nras is particularly relevant to melanoma as Nras mutations are found in 15%–20% of melanoma tumors (Curtin et al., 2005; Jakob et al., 2012). We therefore examined Ras palmitoylation in melanoma tumor arrays using a pan-Ras antibody. The array of strictly melanoma samples allowed comparison between multiple tumor samples with varying levels of ABE-PLA puncta formation (Figure 5B). Quantifying the number of puncta in 3–5 different fields in each tumor core provides insight into variations in background signal in the -HAM control relative to the +HAM treated samples (Figure 5C). We found there were different levels of background between samples as well as within the same sample. For example, sample B8 had very low background (less than 25 puncta/field) relative to the high levels produced by HAM treatment (100–150 puncta/field). In contrast, the background for sample A5 ranged from 30–50 puncta/field, with HAM treated samples generally having 2-fold more puncta than the background. Finally, in core D6 two -HAM fields had about 60 puncta per field with the corresponding fields in the +HAM sample showing only a modest increase (75–80 puncta/field) over the negative control, whereas other fields had similar background levels but much higher puncta in the +HAM condition (120–140 puncta per field). The variability in background between samples could be caused by differences in the quality of individual tissue samples and how samples were handled prior to array preparation.

Overall, these results provide a tantalizing glimpse of the potential for using the ABE-PLA assay as a diagnostic tool or as a biomarker for palmitoylation inhibitors *in vivo* in future, highlighting the power of chemical imaging approaches.

## Conclusion

These are the first reported methods for detecting S-palmitoylated proteins in FFPE tissue samples. Moreover, specific protein targets can also be detected by performing PLA after the ABE assay. Using this strategy, we were able to detect palmitoylated phospho-EGFR in multiple tissue types and palmitoylated Ras in human melanoma samples. This approach could not only provide important biological insights but also represents a conceptual advance in chemical proteomics and PTM specific imaging. The applications of using chemical probes in tissue arrays go far beyond detecting specific palmitoylated proteins. One can envision myriad applications from drug target engagement studies in clinical trials to unbiased screening of chemical probes across tissues to identify tissue specific liabilities.

Our previous studies found that EGF stimulation increased EGFR palmitoylation and that palmitoylated EGFR was also phosphorylated on tyrosine 1068 (Runkle et al., 2016). Detection of palmitoylated EGFR by the conventional ABE assay required cells from a 60 mm dish (approximately 1 × 10^6^ cells) (Fredriksson et al., 2002; Runkle et al., 2016). The ABE-PLA assay can detect EGFR palmitoylation in single cells and detects the increase in dually palmitoylated-phosphorylated EGFR in response to EGF stimulation. Further work is needed to establish the range limits of the assay in detecting palmitoylation before it can be used to correlate palmitoylation of specific proteins with disease state or outcome. Our future work will focus on extending this approach to additional post-translational modifications with additional chemistries and multiplexing multiple PTMs within the same tissue arrays.

## Materials and methods

### Cell culture and drug treatment

NCI-H1975 cells (ATCC) were maintained in RPMI media supplemented with 10% FBS. Cells were treated with 2-bromopalmitate (100 nM) overnight for approximately 15 h.

### ABE on cultured cells

NCI-H1975 cells were serum starved in serum free media for 2 h followed by EGF (100 ng/mL) stimulation for 5 min. Cells were processed using the ABE assay as described previously (Runkle et al., 2016).

### ABE on FFPE mouse tissues

Formalin fixed paraffin embedded mouse tissue samples were sectioned at 4-μm thickness and deparaffinized in xylene and rehydrated with a series of ethanol washes. Antigen retrieval was performed using edetic acid buffer (Electron Microscopy Sciences #62706–11). Immediately after antigen retrieval, samples were incubated in in NEM buffer (50 mM NEM, 150 mM NaCl, 50 mM Hepes pH 7.4, and 10 mM EDTA, and 0.2%Triton X-100) at room temperature, in the dark, for 30 min. The arrays were then washed and re-incubated in fresh NEM buffer overnight at 4°C. Samples were washed in ABE buffer (150 mM NaCl, 50 mM Hepes pH 7.4, and 10 mM EDTA, and 0.2%Triton X-100), then incubated at room temperature for 2 h in the same buffer, with or without 0.7 M hydroxylamine. After washing in ABE buffer, samples were incubated with ABE buffer with added biotin-HPDP (10 µM) for 1 h at room temperature. Samples were then washed in TBST buffer (50 mM Tris HCl pH 7.4, 150 mM NaCl, 0.05% Tween) and then blocked with 5% BSA dissolved in TBST for 1 h at room temperature. Samples were stained by overnight incubation at 4°C with antibodies against biotin (1:1000; abcam), followed by detection with Alexa 488 nm and Alexa 594 nm conjugated secondary antibodies (1:1000; Life Technologies). Coverslips were mounted and DNA staining done using Fluoro-Gel II (EMS #17985–50).

### ABE-PLA on cultured cells

NCI-H1975 cells were grown on 18 mm cover glasses in a 12-well tissue culture plate for 24 h followed by incubation in serum free RPMI media for 80 min, stimulated with EGF (100 ng/mL) for 5 min, then fixed in 10% neutral buffered formalin (Fisher Scientific 23–245-685) containing 50 mM NEM for 30 min at room temperature. After washing in Dulbecco’s phosphate-buffered saline (DPBS), cells were permeabilized in NEM buffer for 30 min at room temperature. Samples were washed in ABE buffer followed by incubation in 4 mM iodoacetamide in ABE buffer for 30 min in the dark at room temperature. After washing, this treatment was repeated with fresh iodoacetamide buffer overnight at 4°C. Samples were washed with ABE buffer, then treated with 0.7 M hydroxylamine as described above for 1 h, then washed once with ABE buffer followed by three washes with DPBS. Next, the samples were incubated in a solution of 10 µM iodoacetamide-alkyne in DPBS for 1 h at room temperature in the dark, followed by washing in DPBS. Cells were then treated with a click reaction mix containing 25 µM OG 488 Azide (Click Chemistry Tools #1264–1), 1 mM CuSO4, and 1 mM tris(2-carboxyethyl) phosphine (TCEP) in DPBS for 45–60 min at room temperature in the dark. After washing in DPBS, proteins were blocked with Duolink blocking solution for 1 hour at 37°C. Incubation with primary antibodies specific for phosphorylated EGFR (Y1068) (Novus Biologicals #MAB3570) and for fluorescein/OG 488 (Life Technologies A-889) was done overnight at 4°C. The Duolink assay was performed as per manufacturer’s protocol with the exception that the amplification time was shortened from 100 min to 60 min.

### ABE-PLA on tissue arrays

Human tissue arrays were purchased from US Biomax, Inc. Slides were heated at 60°C for 4 min, then deparaffinized and rehydrated as described above. Antigen retrieval was performed using citrate buffer (Electron Microscopy Sciences #62706–13). Immediately after antigen retrieval, the tissue array was washed with ABE buffer and then incubated in NEM buffer at 37°C for 30 min. Next, samples were washed three times in ABE buffer, followed by incubation in ABE buffer with added iodoacetamide (4 mM) overnight in the dark at room temperature. The arrays were washed and then re-incubated with fresh ABE buffer containing 4 mM iodoacetamide for 30 min. The samples were washed three times in ABE buffer followed by 1 hour incubation with ABE buffer containing hydroxylamine (0.7 M) as described above. The samples were washed one time in ABE buffer and the 1-h hydroxylamine step was repeated. The arrays were then washed once in ABE buffer followed by three washes in DPBS. The samples were incubated with DPBS containing 10 µM iodoacetamide-alkyne, followed by incubation with click reaction mix as described above, then blocked in 5% BSA in TBST. The remaining steps were as previously described for ABE-PLA on cultured cells. Ras was detected in melanoma tissues using a pan-Ras monoclonal antibody (Invitrogen #PIMA1012X).

### Microscopy

The samples were imaged using a Leica DMI600B widefield epifluorescent microscope. Images were captured using a Hamamatsu Orca-R2 digital camera. A z-series of focal planes were captured and deconvolved using Leica LAS X 3D-deconvolution software.

### Statistics

Statistical analyses were performed using Prism software (GraphPad Prism 9). Experiments are reported as mean ± sd as noted in the legends. Data were analyzed using a 2-tailed Student’s *t*-test for comparison between 2 data sets. A *p*-value of less than 0.05 was considered statistically significant.

## Data Availability

The raw data supporting the conclusion of this article will be made available by the authors, without undue reservation.
