# Erratum: Chemical probe mediated visualization of protein S-palmitoylation in patient tissue samples

**DOI:** 10.3389/fphys.2023.1208618

**Published:** 2023-05-03

**Authors:** Frontiers Production Office

**Affiliations:** Frontiers Media SA, Lausanne, Switzerland

**Keywords:** palmitoylation, cancer, signaling, chemical probe, EGFR

Due to a production error, labels for Figure 3 (c and d) were not included in the final article. The corrected figure appears below.

**FIGURE 3:**
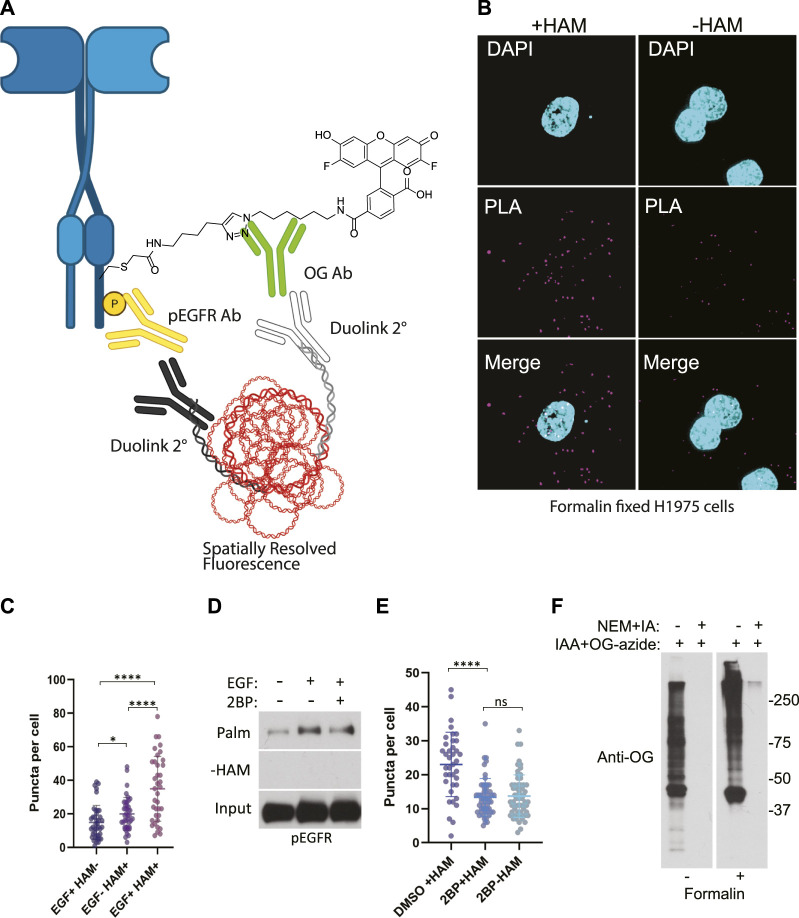
**(A)** Schematic of the ABE-PLA showing method for detecting palmitoylated EGFR in formalin fixed cells processed with the ABE protocol and palmitate was replaced with Oregon green-iodoacetamide. Following the ABE, the proximity ligation assay was performed using primary antibodies to EGFR-phosphoTyrosine1068 and the Oregon Green (OG) label. Samples were incubated with species specific secondary PLA antibodies followed by annealing to single stranded circular DNA which is then amplified with DNA polymerase. The amplified DNA is detected with fluorescently tagged complimentary oligonucleotides and is visualized as fluorescent puncta. **(B)** Formalin fixed H1975 lung cancer cells processed with the ABE-PLA. PLA signal is detected in cells treated with hydroxylamine (+HAM). Omitting hydroxylamine reduces the number of puncta in the negative control (-HAM) DAPI is shown in cyan and PLA signal in magenta. **(C)** Puncta per cell were quantified in all conditions (Unpaired Student’s *T*-test: EGF + HAM-vs. EGF + HAM+ ****p < 0.0001; EGF + HAM + vs. EGF- HAM+ ****p < 0.0001; EGF + HAM-vs EGF-HAM+ *p < 0.05). Total Number of Cells = 119. **(D)** Standard ABE assay of H1975 cells treated with 100 nM 2-bromopalmitate followed by 100 ng/mL EGF stimulation. **(E)** Quantitation of ABE-PLA puncta of cells with or without 2-bromopalmitate with and without EGF stimulation. [Unpaired Student’s *T*-test: DMSO + HAM + vs. 2BP + HAM+ ****p < 0.0001; 2BP + HAM + vs. 2BP + HAM-not significant (ns)]. Total Number of Cells = 153. **(F)** Validation of efficient cysteine blocking and labelling of formalin fixed cells *in vitro*.

The publisher apologizes for this mistake. The original version of this article has been updated.

